# Bone morphogenetic protein 4 (*BMP4*) loss-of-function variant associated with autosomal dominant Stickler syndrome and renal dysplasia

**DOI:** 10.1038/s41431-018-0316-y

**Published:** 2018-12-19

**Authors:** Thomas R. W. Nixon, Allan Richards, Laura K. Towns, Gavin Fuller, Stephen Abbs, Philip Alexander, Annie McNinch, Richard N. Sandford, Martin P. Snead

**Affiliations:** 1 School of Clinical Medicine, University of Cambridge, Addenbrooke’s Hospital, Hills Road, Cambridge, CB2 0SP UK; 2 Vitreoretinal Service, Addenbrooke’s Hospital, Hills Road, Cambridge University Hospitals NHS Foundation Trust, Cambridge, CB2 0QQ UK; 3 Department of Pathology, University of Cambridge, Tennis Court Road, Cambridge, CB2 1QP UK; 4 East Anglian Medical Genetics Service, Box 134, Addenbrooke’s Treatment Centre, Hills Road, Cambridge, CB2 0QQ UK

**Keywords:** Disease genetics, Genetics research, Disease genetics

## Abstract

Stickler syndrome is a genetic disorder that can lead to joint problems, hearing difficulties and retinal detachment. Genes encoding collagen types II, IX and XI are usually responsible, but some families have no causal variant identified. We investigate a variant in the gene encoding growth factor BMP4 in a family with Stickler syndrome with associated renal dysplasia. Next generation sequencing of the coding region of *COL2A1*, *COL11A1* and a panel of genes associated with congenital anomalies of the kidney and urinary tract (CAKUT) was performed. A novel heterozygous *BMP4* variant causing a premature stop codon, c. 130G>T, p.(Gly44Ter), which segregated with clinical features of Stickler syndrome in multiple family members, was identified. No variant affecting gene function was detected in *COL2A1* or *COL11A1*. Skin fibroblasts were cultured with and without emetine, and the mRNA extracted and analysed by Sanger sequencing to assess whether the change was causing nonsense-mediated decay. Nonsense-mediated decay was not observed from the extracted *BMP4* mRNA. *BMP4* is a growth factor known to contribute to eye development in animals, and gene variants in humans have been linked to microphthalmia/anophthalmia as well as CAKUT. The variant identified here further demonstrates the importance of BMP4 in eye development. This is the first report of a *BMP4* DNA variant causing Stickler syndrome, and we suggest *BMP4* be added to standard diagnostic gene panels for this condition.

## Introduction

Stickler syndrome is a genetic disorder affecting the eyes, joints, hearing and orofacial development. It is associated with a significantly increased lifetime risk of rhegmatogenous retinal detachment and is the most common cause of non-traumatic retinal detachment in children. Other ocular features include myopia, often severe, and congenital defects of the vitreous, which can be a useful diagnostic feature and helps direct genetic testing [1, 2]. Hearing loss, present in 63% of patients, is usually high frequency sensorineural loss, with a variable conductive component in children due to intermittent otitis media [3]. Joint problems, in 75%, include hypermobility and premature osteoarthritis leading to chronic back pain and early joint replacement surgery [4]. Orofacial manifestations include cleft palate (41%), Pierre Robin sequence (24%) and midfacial hypoplasia [5, 6]. There is, however, marked variability in the degree to which each system is affected in different patients [6].

Most cases of Stickler syndrome are due to variants in genes encoding various fibrillar collagens. Type 1 Stickler syndrome is the most common, accounting for ~80% of the cases, and is due to heterozygous changes in *COL2A1* (ref. 2), which encodes the alpha-1 chain of type II collagen. It carries the greatest risk of retinal detachment, but generally milder hearing loss. Type 2 Stickler syndrome is caused by heterozygous alteration to *COL11A1* (ref. 7), which encodes the alpha-1 chain of type XI collagen. Type 3 Stickler syndrome is caused by heterozygous changes to *COL11A2* (ref. 8), which is not expressed in the eye, resulting in a non-ocular phenotype. There is an ocular-only variant caused by variants in *COL2A1* affecting exon 2 (ref. 9), which is only expressed in the eye. Less frequently, recessive Stickler syndrome has been found to be caused by homozygous variants in *COL9A1* (ref. 10), COL9A2 (ref. 11) and *COL9A3* (ref. 12), and bi-allelic *COL11A1* sequence changes [13].

Stickler syndrome has been associated with recessive variants in two non-collagen genes: one family with a homozygous variant in *LOXL3* (ref. 14), which encodes an enzyme involved in collagen cross-linking and one family with a homozygous variant in *LRP2* (ref. 15), encoding an endocytic transmembrane receptor.

The bone morphogenetic proteins (BMPs) are growth factors that are part of the TGF-β superfamily [16]. They are expressed throughout embryogenesis and have diverse roles in development. Initially identified and named for their role in osteogenesis, they are now recognised to have a crucial role in early embryonic development in gastrulation and neurulation, mesoderm patterning, and cell proliferation, differentiation and morphogenesis in many organ systems. BMP4 is known to have a critical role in ocular development in establishing the dorsal-ventral axis of the developing eye [17] as well as lens induction and retinal development [18]. Humans with loss-of-function variants in *BMP4* have previously been described with highly variable manifestations, including anophthalmia/microphthalmia, syndactyly and structural brain anomalies including thinning of the corpus callosum and widened cerebral sulci [19, 20]. Deletions of the 14q22-q23 region, which includes *BMP4* as well as *OTX2*, also known to be important for eye development, have been described causing anophthalmia/microphthalmia and also hearing problems, Pierre-Robin sequence, developmental delay and cleft palate [21–23]. Mice models with *bmp4* loss-of-function variants have features including microphthalmia, craniofacial anomalies, polydactyly and congenital anomalies of the kidney and urinary tract (CAKUT) [24].

In this study, we investigate a family with clinical features of Stickler syndrome, but in which one member of the family also had congenital renal dysplasia leading to renal replacement therapy at a young age. Next generation sequencing identified a sequence variant in the gene for *BMP4*, which segregated with the Stickler syndrome phenotype in this family.

### Patient data

**Fig. 1:**
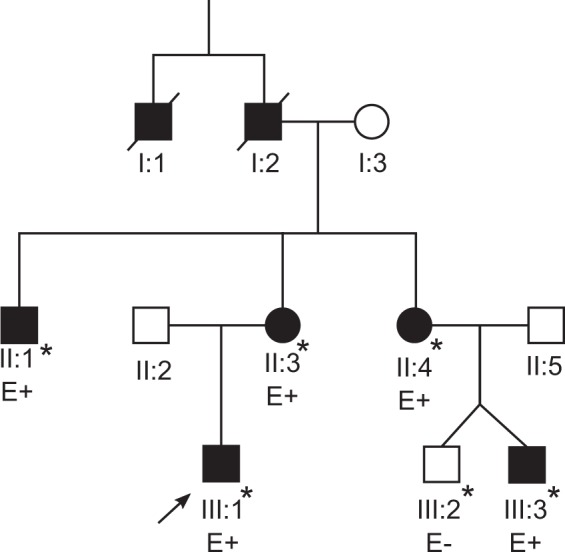
Family pedigree. Patients clinically affected are in solid. * Ophthalmologically evaluated clinically (Martin Snead and Thomas Nixon). E+ DNA evaluated as positive for familial BMP4 variant. E− DNA evaluated as negative for familial BMP4 variant

**Fig. 2:**
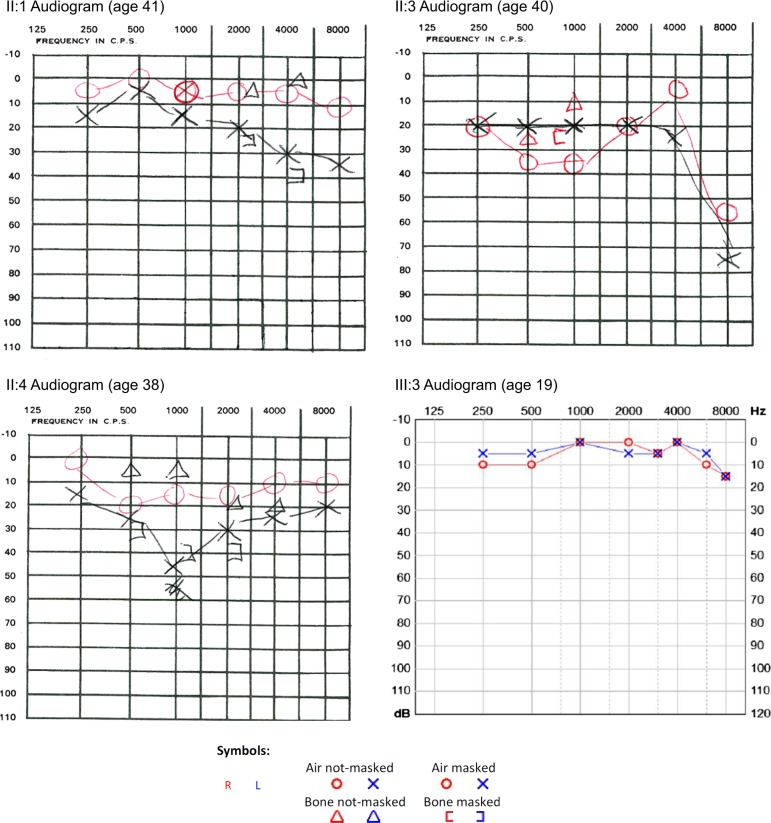
Audiograms. Pure tone audiometry thresholds for patients II:1, II:3, II:4 and III:3

**Fig. 3:**
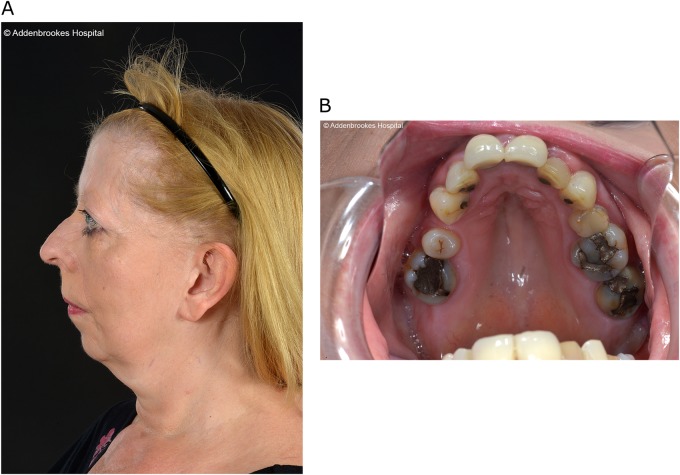
Clinical photographs of patient II:3. **a** Right lateral facial photograph of patient II:3 demonstrating retrognathia. **b** Intra-oral photograph of patient II:3 demonstrating high-arched palate

One male, aged 20, with renal dysplasia requiring renal replacement therapy was referred to clinical genetics (Patient III:1 in Fig. 1, Table 1). It was noted that several family members had a clinical diagnosis of Stickler syndrome, with five affected members over two generations (Patients I:1, I:2, II:1, II:3, II:4 in Fig 1, Table 1). Previous sequencing of the *COL2A1* and *COL11A1* genes in one of the affected family members had not identified a variant that would affect function. The clinical features present in the family included high myopia, congenital hypoplasia of the vitreous, retinal detachment, sensorineural hearing loss (Fig. 2), retrognathia and high-arched palate (Fig. 3). As a result, this patient was referred to the National Stickler syndrome diagnostic service, which confirmed that he also had myopia with abnormal vitreous architecture. Two further family members were examined (III:2 and III:3), of which one had myopia with abnormal vitreous and the other had normal ophthalmological examination. No other family members reported kidney disease. Renal ultrasound was offered but was declined.

**Table 1:** Table of affected family members (Fig. 1)

Clinical features	Eye	Hearing	Joint	Other
III:1	Myopia (−4.5D/−2.5D), hypoplastic vitreous	No hearing loss	No problems	Renal dysplasia, high palatal notch
III:3	Myopia (−5D/−5D), hypoplastic vitreous	Mild high frequency sensorineural hearing loss	No problems	Flat mid face
II:1	Myopia, bilateral retinal detachment, first at age 17	Sensorineural hearing loss	No problems	High-arched palate, flat midface, retrognathia
II:3	Myopia (−16D/−15D), bilateral retinal retinal detachment, first at age 40	High frequency sensorineural hearing loss requiring hearing aids at 50	Hypermobility	High-arched palate, retrognathia
II:4	Myopia (−18D), bilateral retinal detachment, first at age 27	Sensorineural hearing loss	No problems	High-arched palate, flat midface, retrognathia
I:1	Myopia, blind in one eye, cause unknown	Unknown	Unknown	Unknown
I:2	Poor eyesight, unknown eye problem	Unknown	Unknown	Unknown

Clinical features of the family described in this paper. Patients in generations II and III all share the familial c.130G>T BMP4 variant. Patients in generation I are deceased: information was gathered from relatives, but no clinical records or DNA were available for evaluation

## Methods

### Genetic analysis

Next generation sequencing was performed using the TruSight One sequencing kit (Illumina, Cambridge, United Kingdom). Variants in a panel of genes known to cause CAKUT (see supplementary information) were identified. 99.69% of the target sequence within this panel was sequenced to a depth of 20-fold or more, with analytical sensitivity of 98.3%–100% (95% Confidence Intervals). Further analysis identified a heterozygous loss-of-function variant in the *BMP4* (NM_001202.3) gene: c.130G>T, p.(Gly44Ter). DNA variant analysis conformed to best practice guidelines [25]. Sanger sequencing was used to confirm the sequence change and to analyse additional family members. These variants were submitted to the LOVD database and can be found at www.LOVD.nl/BMP4 (patient IDs 155750, 165221, 165222, 165224 and 165225).

### mRNA analysis

RNA was extracted using RNeasy column, (Qiagen, Manchester, United Kingdom) from cultured dermal fibroblasts (family member II-3), that had been incubated either with or without emetine, an inhibitor of nonsense-mediated decay (as previously described) [26]. Reverse-transcription PCR was performed using Superscript II Reverse Transcriptase (Invitrogen, Hemel Hempstead, United Kingdom) and an antisense primer: 5′-TGTAGTGTGTGGGTGAGTGGATGG-3′. PCR amplification was then performed of a 940 bp region of the *BMP4* cDNA surrounding the variant, using Q5 High-fidelity DNA polymerase (NEB, Hitchin, United Kingdom) in reaction conditions as recommended by the manufacturer. A primary reaction used primers 5′-ATGATTCCTGGTAACCGAATGCTG-3′ in exon 3, and 5′-ACCTTATCATACTCATCCAGGTAC-3′ in exon 4. Products from this primary reaction were then re-amplified in a secondary reaction using primers 5′-ATTATGCCAAGTCCTGCTAGGAGG-3′ in exon 3, and 5′-GGGCCACAATCCAGTCATTCCAGC-3′ in exon 4. The resulting cDNA product was sequenced using the primer 5′-ATGTTCTTCGTGGTGGAAGCTC-3′ using standard methods for Sanger sequencing.

## Results

### Sequencing

**Fig. 4:**
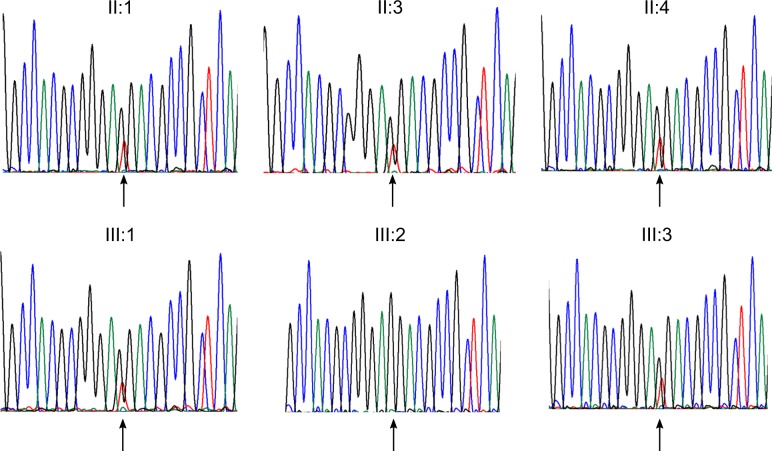
Sequencing chromatographs for tested family members. Chromatographs demonstrating a heterozygous G>T BMP4 substitution (arrowed) in affected family members, with no substitution (arrowed) in unaffected family member (III:2)

Next generation sequencing of the patient identified a heterozygous sequence change in the *BMP4* gene: NM_001202.3: c.130G>T, p.(Gly44Ter). This is predicted to cause a premature termination codon. Sanger sequencing confirmed that this variant was present in family members with the Stickler syndrome phenotype and not present in those with a normal phenotype (Fig. 4). The sequence change was not present in public DNA sequence variation databases (http://exac.broadinstitute.org/; http://www.internationalgenome.org/).

### mRNA analysis

**Fig. 5:**
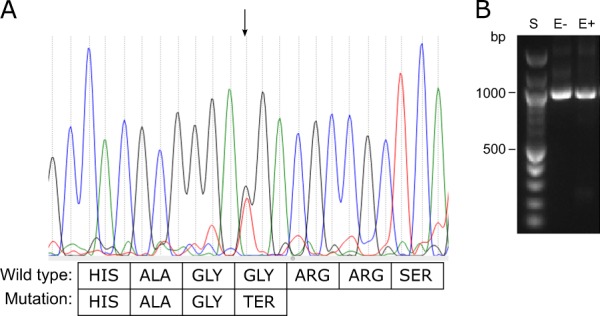
Sequencing chromatograph from RT-PCR. **a** Sanger sequencing of RT-PCR products (sense orientation) demonstrating heterozygosity in mRNA extracted from untreated (- emetine) cultured dermal fibroblasts at the variant locus, i.e. nonsense-mediated decay does not take place. The G>T variant (arrowed) changes a GGA coding a glycine residue into a premature stop codon. **b** Agarose gel of PCR products. S size ladder. E− sample from cells cultured without emetine, E+ sample from cells cultured with emetine. PCR products are of expected cDNA size of ~1 kb

Sanger sequencing of the RT-PCR products demonstrated that both variant and wild-type sequences were present (Fig. 5) demonstrating that the variant did not result in nonsense-mediated decay, but would result in a severely truncated protein.

## Discussion

We have described a novel variant in a gene, *BMP4*, associated with a Stickler syndrome phenotype. The vast majority of patients with dominant Stickler syndrome are found to have *COL2A1* or *COL11A1* variants [2], and in our experience less than 5% of patients that have a clear clinical diagnosis have no detectable variants that affect function in either of these two genes, or those encoding type IX collagen. This is the first description of a *BMP4* gene variant associated with a clinical phenotype of Stickler syndrome and is the first non-collagen sequence change found to be associated with dominant Stickler syndrome, although recessive Stickler or Stickler-like syndromes have been described in two other non-collagen genes, *LOXL3* (ref. 14) and *LRP2* (ref. 15). One consanguineous family with two children diagnosed with Stickler syndrome, cleft palate, micrognathia and congenital high myopia were found to have a homozygous change in *LOXL3*, which encodes the enzyme lysyl oxidase-like 3. Lysyl oxidase-like 3 is known to cross-link type II collagen, which underlines the fundamental collagenopathy in Stickler syndrome. A further consanguineous family with two children diagnosed with Stickler syndrome, with congenital high myopia, hypoplastic vitreous, a possible vitreous membranous anomaly similar to type 1 Stickler syndrome, giant retinal tear detachment and joint hypermobility, were found to have a homozygous change in *LRP2*. *LRP2* encodes lipoprotein receptor-related protein-2, an endocytic transmembrane receptor. Variants in *LRP2* are known to cause Donnai–Barrow syndrome, a rare autosomal recessive disorder featuring agenesis of the corpus callosum, sensorineural hearing loss, facial dysmorphism and ocular findings, including myopia and coloboma. The pathway to cause Stickler syndrome is less clear in this gene, but it likely interferes with intercellular signalling during embryogenesis.

Like other members of the TGF-β superfamily, BMP4 is synthesised as a precursor molecule and is proteolytically cleaved into two domains, an N-terminal prodomain, (which can vary in length due to different transcripts) and a C-terminal active growth factor dimer forming region [27]. As well as homodimers, BMP4 also forms heterodimers with BMP7, with the heterodimers being more active [28]. Heterozygous *BMP7* loss-of-function variants have also been associated with anophthalmia [29].

**Fig. 6:**
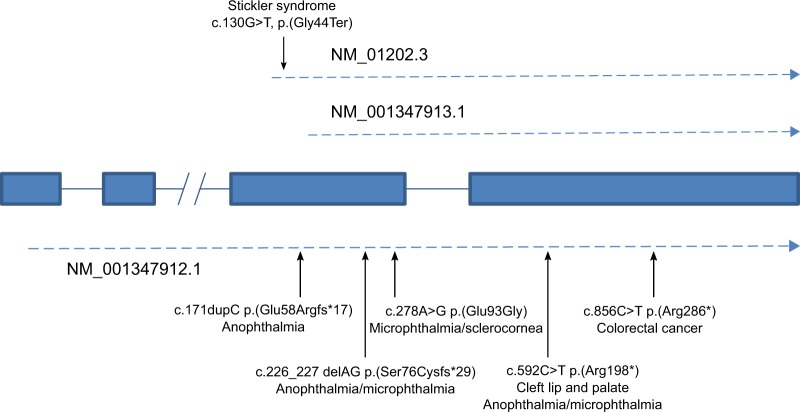
BMP4 transcripts and variant positions. The *BMP4* gene consists of four exons. Transcription can start at multiple locations on the gene. All variants are described relative to their position on transcript NM_01202.3. Three different BMP4 transcripts are shown, demonstrating the position of the c.130G>T variant compared with those previously described. Transcript NM_001347913.1 is unaffected by this variant. All previously described variants are downstream of the variant described in this paper and all affect the NM_001347913.1 transcript, except c.171dupC

With a 24 amino acid signal peptide, the c. 130G>T, p.(Gly44Ter) [transcript variant NM_001347912.1; c.271G>T, p.(Gly91Ter)] variant we describe here results in only 19 (or 66 from the longer transcript, Fig. 6) amino acids of the BMP4 prodomain being expressed (the mRNA does not undergo nonsense-mediated decay). This may explain why, when compared with previously described *BMP4* variants, the ocular phenotype in this family is mild. The majority of documented *BMP4* loss-of-function variants have severe ocular anomalies, including anophthalmia, microphthalmia, and anterior chamber abnormalities with congenital glaucoma [19–23]. Systemic features are variable and include developmental delay, brain and pituitary anomalies, craniofacial dysmorphism, kidney anomalies and polydactyly. Those previously described variants associated with disease were downstream of the variant in the pedigree described here. It is possible, therefore, that those changes produced an abnormal protein with a dominant negative effect, not present in this pedigree, which in this case may simply reflect haploinsufficiency of BMP4 (due to lack of functional protein from severe truncation). Interestingly the c.171dupC, p.(Glu58Argfs*17) [NM_001347912.1; c.312dupC, p.(Glu105Argfs*17)] *BMP4* variant only encodes an additional 14 normal amino acids of the prodomain yet that change results in a severe ocular phenotype [20]. BMP4 prodomains can form a stable interaction with the active domains and alter their affinity with extracellular matrix molecules [28]. The difference in resulting phenotypes between c. 130G>T, p.(Gly44Ter) and c.171dupC, p.(Glu58Argfs*17) may therefore reflect the ability of these truncated prodomains to form a complex with growth factor dimers and produce a dominant-negative effect. It is interesting to contrast the microphthalmia/anophthalmia seen in previously described *BMP4* variants with the megalophthalmos and myopia seen in this pedigree.

Another mechanism that potentially modifies the phenotype, which needs to be considered is that not all *BMP4* transcripts contain the variant described here (Fig. 6). Transcripts NM_001347913.1, NM_001347915.1, NM_001347917.1, all have the initiating methionine downstream of the c.130G>T variant and do not encode the first 63 amino acids of the NM_01202.3 transcript. However, this is also true for the c.171dupC p.(Glu58Argfs*17) variant and so is unlikely to be a major consideration.

A number of cases involve microdeletions, including BMP4 and adjacent genes in the 14q22–23 region. *OTX2* is only 2.8 Mbs from *BMP4* and is also critical in eye development, with variants also known to cause microphthalmia/anophthalmia. Concurrent deletions of both genes have been associated with microphthalmia/anophthalmia with decreased cerebral white matter volume [30].

BMP4 has been shown to be critical in establishing the dorsal-ventral axis of the developing eye [17]. In the chick eye, *bmp4* is expressed in the dorsal optic cup and is responsible for spatiotemporal co-ordination of apoptotic cell death [31]. In *Xenopus*, bmp4 has been shown to act via inducing tbx5 to determine the dorsal region of the optic cup, with the factor sonic hedgehog having an opposing effect [17]. In mouse embryos, increased levels of bmp4 were shown to reduce retinal volume, alter the shape of the optic cup and reduce axial length—this suggests that low levels of BMP4 could contribute to increased axial length and therefore congenital myopia [32]. BMP4 is also critical for lens induction [19].

The interaction between BMP4 and TGFβ1 may be important in its role in pathogenesis of retinal detachment. It is known that exon 2 of *COL2A1*, expressed in the eye and developing cartilage but not mature cartilage, encodes a region of type II procollagen with a cysteine-rich domain that binds TGFβ1 and BMP2 (ref. 33). Loss-of-function variants in *COL2A1* cause type 1 Stickler syndrome, with vitreous anomaly and significant risk of retinal detachment. Additionally, intronic sequence variants affecting the alternative splicing efficiency of exon 2 of *COL2A1* have been associated with an increased risk of retinal detachment [34]. BMP4 and BMP2 have significant structural similarity so it is possible that BMP4 can also bind to type II collagen [35]. TGFβ1 has also been shown to bind to type IV collagen [36]. This is an important structural component of the inner limiting membrane of the retina, which then contributes to the posterior hyaloid membrane of the vitreous [37], which if too adherent to the retina can cause a retinal tear leading to retinal detachment. Fibrillin is a major component of microfibrils in extracellular matrices, and variants in the fibrillin gene (*FBN1*) can cause Marfan syndrome, which has many features including myopia and risk of retinal detachment. It has been shown that mice deficient in fibrillin have dysregulation of TGFβ1 (ref. 38). It has also been demonstrated that fibrillin assemblies have a role in directing signalling through TGFβ1 and BMP pathways [39]. This may suggest that TGFβ1 dysregulation may be a ‘final common pathway’ in the pathogenesis of retinal detachment in patients with a genetic predisposition.

The c.130G>T *BMP4* variant was initially identified in the proband through testing a panel of genes known to be associated with CAKUT. No other family members are known to have renal disease but detailed imaging has not been carried out in all individuals who have the *BMP4* variant. The aetiology of CAKUT is complex. A family history of CAKUT is found in about 10 to 20% of cases but only a minority will have an identifiable genetic cause identified. Where a genetic cause is identified, the pattern of inheritance and segregation of the disease phenotype typically appears as an autosomal dominant trait with incomplete penetrance and variable expression. This marked variability, also seen in animal models of CAKUT, may be due to a number of different factors including hypomorphic variants that do not completely disrupt early nephrogenesis but affect renal and urinary tract development at later stages of gestation and genetic or environmental modifiers [40].

In summary, a *BMP4* variant has been identified as a cause of autosomal dominant Stickler syndrome. Molecular genetic diagnosis is important for families to allow appropriate follow-up, risk management advice and genetic counselling regarding family planning, so testing for *BMP4* should be considered in Stickler syndrome where no variant is identified in a collagen gene that would be expected to affect function. If a loss-of-function *BMP4* variant is identified in a patient with Stickler syndrome then patients should be investigated for CAKUT, and conversely if identified in a patient with CAKUT, they should be referred for ophthalmological assessment.

## Supplementary information

Supplemental material
