# Supplementary material for: Bone morphogenetic protein 4 (BMP4) loss-of-function variant associated with autosomal dominant Stickler syndrome and renal dysplasia
Source: Eur J Hum Genet. 2018 Dec 19;27(3):369–77. doi: 10.1038/s41431-018-0316-y (PMC6460578; doi:10.1038/s41431-018-0316-y)
Supplement: Supplementary file 1 — Supplemental material [file 41431_2018_316_MOESM1_ESM.docx]

Supplementary information: CAKUT gene panel

List of all genes screened in the CAKUT (congenital anomalies of the kidney and urinary tract) with NCBI accession number

ACE ( NM_000789.3 ); ACVR2B ( NM_001106.3 ); AGT ( NM_000029.3 ); AGTR1 ( NM_000685.4 ); AGTR2 ( NM_000686.4 ); ALDH1A2 ( NM_003888.3 ); ALK ( NM_004304.4 ); BICC1 ( NM_001080512.1 ); BMP4 ( NM_001202.3 ); BMP5 ( NM_021073.2 ); BMP7 ( NM_001719.2 ); BMPER ( NM_133468.4 ); CHD1L ( NM_004284.4 ); DHCR7 ( NM_001360.2 ); DLX5 ( NM_005221.5 ); DLX6 ( NM_005222.3 ); EFEMP1 ( NM_001039348.2 ); EMX2 ( NM_004098.3 ); EPHA7 ( NM_004440.3 ); EYA1 ( NM_172058.2 ); FGF10 ( NM_004465.1 ); FGF20 ( NM_019851.2 ); FGFR2 ( NM_022970.3 ); FOXC1 ( NM_001453.2 ); FOXC2 ( NM_005251.2 ); FRAS1 ( NM_025074.6 ); FREM2 ( NM_207361.4 ); GATA1 ( NM_002049.3 ); GATA2 ( NM_032638.4 ); GATA3 ( NM_001002295.1 ); GDNF ( NM_001190468.1 ); GFRA1 ( NM_005264.4 ); GPC3 ( NM_001164617.1 ); GREM1 ( NM_013372.6 ); GRIP1 ( NM_021150.3 ); HNF1B ( NM_000458.2 ); HOXA11 ( NM_005523.5 ); ISL1 ( NM_002202.2 ); ITGAM ( NM_001145808.1 ); KAL1 ( NM_000216.2 ); L1CAM ( NM_000425.3 ); LHX1 ( NM_005568.3 ); MUC1 ( NM_001204286.1 ); NBEA ( NM_015678.4 ); NFIA ( NM_001145512.1 ); PAX2 ( NM_003988.3 ); PTCH1 ( NM_000264.3 ); PTGS2 ( NM_000963.2 ); REN ( NM_000537.3 ); RET ( NM_020975.4 ); ROBO2 ( NM_001128929.2 ); SALL1 ( NM_002968.2 ); SALL4 ( NM_020436.3 ); SHH ( NM_000193.2 ); SIX1 ( NM_005982.3 ); SIX2 ( NM_016932.4 ); SIX5 ( NM_175875.4 ); SMAD4 ( NM_005359.5 ); SMARCA4 ( NM_001128849.1 ); SOX17 ( NM_022454.3 ); SOX9 ( NM_000346.3 ); SPRY2 ( NM_005842.2 ); TFAP2A ( NM_003220.2 ); TNXB ( NM_019105.6 ); TP63 ( NM_003722.4 ); UMOD ( NM_003361.2 ); UPK3A ( NM_006953.3 ); VCAN ( NM_004385.4 ); WNT4 ( NM_030761.4 ); WT1 ( NM_024426.4 ); XPNPEP3 ( NM_022098.3 ),
